# Antiviral properties of resveratrol against pseudorabies virus are associated with the inhibition of IκB kinase activation

**DOI:** 10.1038/s41598-017-09365-0

**Published:** 2017-08-18

**Authors:** Xinghong Zhao, Qiankun Cui, Qiuting Fu, Xu Song, Renyong Jia, Yi Yang, Yuanfeng Zou, Lixia Li, Changliang He, Xiaoxia Liang, Lizi Yin, Juchun Lin, Gang Ye, Gang Shu, Ling Zhao, Fei Shi, Cheng Lv, Zhongqiong Yin

**Affiliations:** 1 Natural Medicine Research Center, College of Veterinary Medicine, Sichuan Agricultural University, Chengdu, 611130 China; 2 Key laboratory of Animal Disease and Human Health of Sichuan Province, Sichuan Agricultural University, Chengdu, 611130 China

## Abstract

Pseudorabies virus (PRV) is a pathogen of swine resulting in devastating disease and economic losses worldwide. Resveratrol (Res) exhibits inhibitory activity against a wide range of viruses. Despite these important advances, the molecular mechanism(s) by which Res exerts its broad biological effects have not yet been elucidated. In this paper, the antiviral activity of Res against PRV and its mechanism of action were investigated. The results showed that Res potently inhibited PRV replication in a dose-dependent manner, with a 50% inhibition concentration of 17.17 μM. The inhibition of virus multiplication in the presence of Res was not attributed to direct inactivation or inhibition of viral entry into the host cells but to the inhibition of viral multiplication in host cells. Further studies demonstrated that Res is a potent inhibitor of both NF-κB activation and NF-κB-dependent gene expression through its ability to inhibit IκB kinase activity, which is the key regulator in NF-κB activation. Thus, the inhibitory effect of Res on PRV-induced cell death and gene expression may be due to its ability to inhibit the degradation of IκB kinase. These results provided a new alternative control measure for PRV infection and new insights into the antiviral mechanism of Res.

## Introduction

Pseudorabies virus (PRV), a member of the swine herpesvirus of the *Alphaherpesvirinae* subfamily, is the pathogen for Aujeszky’s disease (AD), which is one of the most devastating infectious diseases in swine and causes enormous economic loss because of its worldwide distribution and high herd mortality^1^. AD is a contagious disease that is characterized by encephalomyelitis and is frequently accompanied by upper respiratory tract inflammation and lung inflammation^2^. In young piglets, PRV infection is often fatal, and piglets die from central nervous system disorders. In contrast, older pigs generally develop respiratory disease. After survival from acute infection, the pigs carry the virus in a latent form for their entire lives. In pregnant sows, PRV infection generally leads to reproductive failure^3, 4^. Despite that vaccines were widely used in controlling PRV, the recombination events resulting from different vaccine strains are important causes of morbidity and mortality^5, 6^.

Natural medications have a wide range of acceptability for the prevention and treatment of diseases throughout history, and they are attracting increasing interest for the development of potential antiviral drugs. Resveratrol (3,5,4-trihydroxystilbene, Res, Figure S1), a non-flavonoid polyphenol compound widely existing in several higher plants, has been reported to have a wide range of bioactivities against many diseases, such as cancer, myocardial infarction, inflammation, immunity, stroke, brain damage, diabetes and viral diseases^7^. The antiviral effect of Res has been demonstrated for a wide range of viruses, including herpesviruses^8–10^, retroviruses^11, 12^, respiratory viruses^13^ and proviral^14, 15^. The effects of Res on the viral life cycle are shown in the supplementary material (Table S1). Despite these important advances, the molecular mechanism by which Res exerts its broad antiviral effects has not yet been elucidated.

As a potential antiviral agent, little is known about the antiviral activity of Res against PRV. Therefore, in the present study, Res was tested for anti-PRV activity, and a molecular mechanism was also elucidated for the purpose of developing a new alternative control measure for PRV infection.

## Results

### Cytotoxicity of Res

The cytotoxicity of Res in duck embryo fibroblasts has previously been tested in our laboratory^16^. We tested a range of concentrations at which Res may exhibit potential cytotoxic activity on PK-15 cells. After treatment for 48 h, the number of viable cells was determined using the CCK-8 kit. Ethanol with a concentration lower than 0.5% (v/v) had no effect on the PK-15 monolayers or PRV proliferation. Throughout the test, the final concentration of ethanol was 0.1% (v/v). Res exhibited cytotoxicity at concentrations of 131.43 and 262.87 μM, and the 50% cytotoxic concentration (50% of cell survival, CC_50_) was above 262.87 μM (Figure S2). Therefore, we selected optimal non-toxic working concentrations (65.72 μM) for antiviral tests.

### Antiviral activity of Res

**Figure 1:**
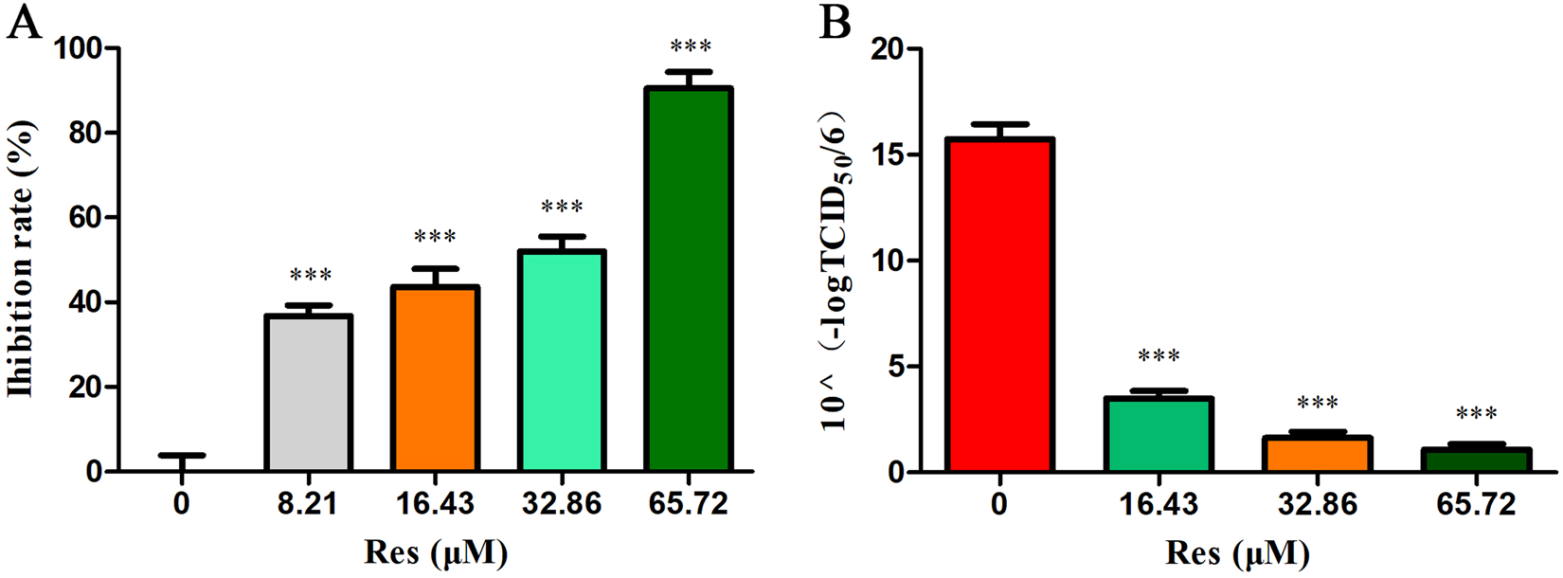
Antiviral activity of Res. The inhibition rate of Res on infection with PRV (**A**): PK-15 cells were first infected with PRV (100TCID_50_) and then treated with Res. After incubation for 48 h, a CCK-8 assay was performed, and the results were expressed as percent of inhibition in drug-treated cultures compared with the untreated group. Viral titres of PRV at growing conditions with different concentrations of Res (**B**): PK-15 cells were infected with PRV (100TCID_50_) and then treated with Res. After incubation for 48 h, the supernatants were collected and analysed for TCID_50_ values. Values are means ± SD (n = 6). Correlation analyses were evaluated by Pearson r2, ns: p > 0.05, *p < 0.05, **p < 0.01, and ***p < 0.001 vs. PRV-infected cells.

Res was assayed for its capability to inhibit PRV multiplication *in vitro* via CCK-8 and TCID_50_ assays, as previously described. PK-15 cells were infected with PRV, and then Res was added. After 48 h, cell viability was evaluated via CCK-8 assay, and virus titres of the culture media were determined via the yield reduction assay. Res showed a significant inhibitory activity against cell death induced by PRV infection in a dose-dependent manner. The inhibition rate was 90.5% at a concentration of 65.72 μM (Fig. 1A). The EC_50_ of Res was estimated to be 17.17 μM, and the selectivity index (CC_50_/EC_50_) was above 15.30 (Table 1). In the virus yield reduction assay, PRV titres in the presence of Res were significantly reduced in a dose-dependent manner. The viral titres were decreased by 77.8%, 89.7% and 93.2% at concentrations of 16.43, 32.86 and 65.72 μM, respectively (Fig. 1B).

**Table 1:** Inhibition effects of Res on PRV *in vitro* ^a^.

Compound	CC_50_ (μM)^b^	EC_50_ (μM)^c^	SI^d^
Res	>262.87	17.17 ± 0.35	>15.30 ± 0.33

^a^The inhibition effects on PRV were evaluated by CCK-8 assay.

^b^Effective concentration 50% (EC50): concentration required to inhibit PRV at 48 h post-infection by 50% (n = 6).

^c^Cytotoxic concentration 50% (CC50): concentration required to reduce cell viability by 50% (n = 6).

^d^SI: the selectivity index is defined as the ratio of CC_50_ to EC_50_.

### Influence of Res on the viral growth curve

PK-15 cells were infected with PRV (MOI = 0.01) in the presence or absence of Res. The total DNA of cells was extracted at the different time points, and the copies of the PRV gene were detected by FQ-PCR assay. The gene copies were calculated according to the standard curve: lg [virus copies] = −0.2843 Cq + 11.902 (R^2^ = 0.996).

**Figure 2:**
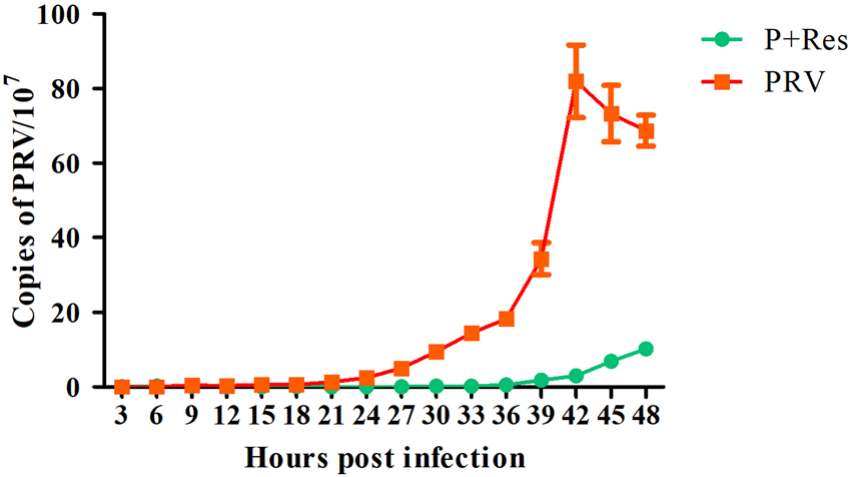
Growth curves of PRV in PK-15 cells in the presence or absence of Res. PK-15 cells were infected with PRV at an MOI of 0.01 and test Res (65.72 μM) solution for 1 h at 37 °C. PRV proliferated in the PK-15 cells in the presence or absence of Res within 48 h. The total DNA was extracted at the indicated time points, and copies of PRV were detected via real-time FQ-PCR. Values are means ± SD (n = 3).

The growth curves of PRV in PK-15 cells in the presence or absence of Res are shown in Fig. 2. Almost no viral proliferation was observed between 0 to 21 hpi. In untreated cells, PRV began to reproduce at 21 hpi, and the number of DNA copies showed a rapid increase within the next 21 h. Copies of the PRV gene decreased at 45 and 48 hpi, which was probably due to the nutrient depletion and cell death caused by virus multiplication. In Res-treated cells, no replication was observed within 36 h, and the number of gene copies slowly increased during the next 12 h, which was significantly less compared with the untreated cells.

### Mode of action

**Figure 3:**
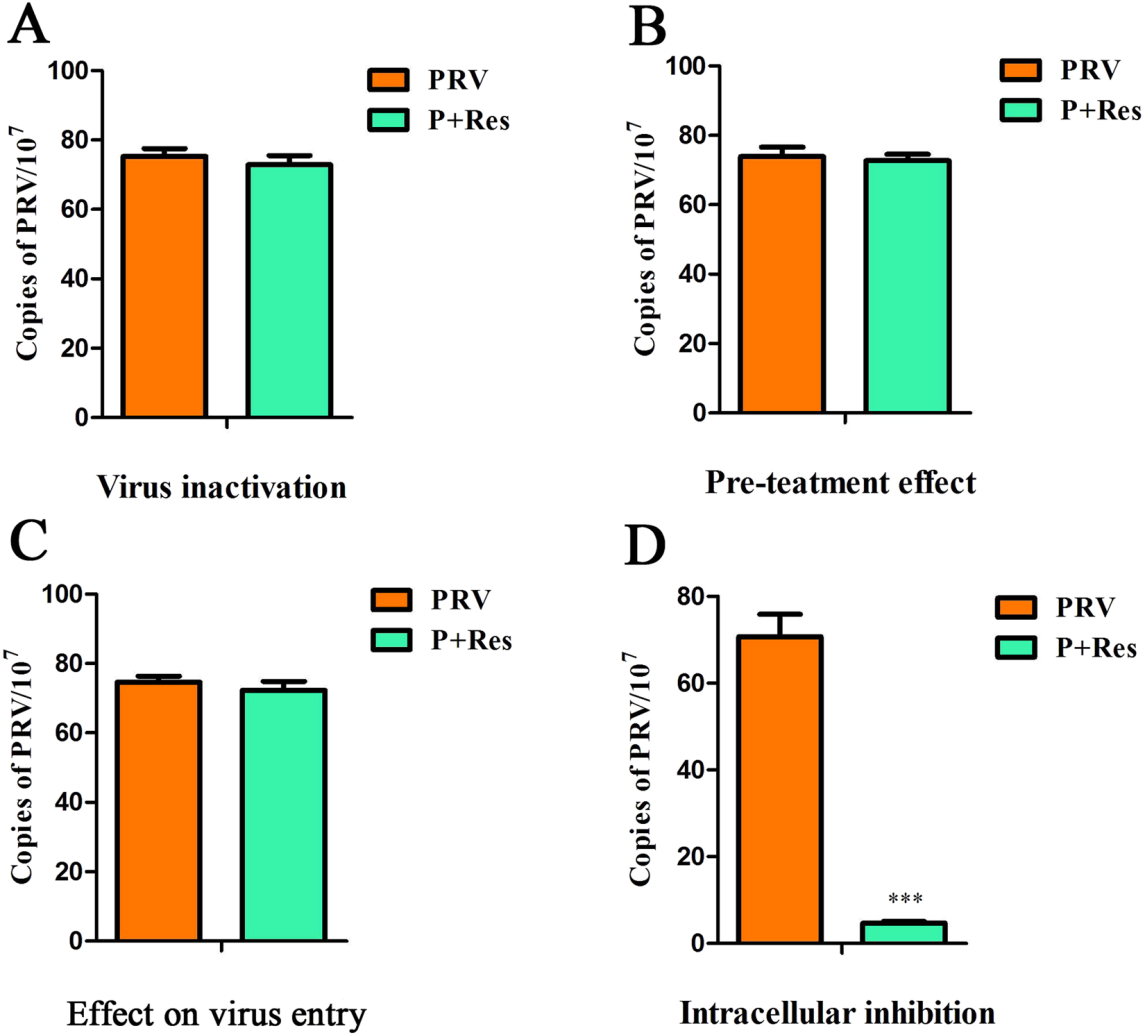
Influence of different treatment conditions of Res on PRV infection. Pk-15 cells infected with PRV (MOI = 0.01) were treated with Res (65.72 μM) under different treatment conditions. Virus inactivation (**A**), Pre-treatment effect (**B**), Inhibition of virus entry (**C**), Intracellular inhibition (**D**). Values are means ± SD (n = 3). Correlation analyses were evaluated by Pearson r2, ns: p > 0.05, *p < 0.05, **p < 0.01, and ***p < 0.001 vs. PRV-infected cells.

Further study of the mode of action was conducted to evaluate which stage was affected by Res during the PRV life cycle. As shown in Fig. 3, the number of PRV gene copies in the replication assay was significantly lower than that of the control group, whereas for the virus entry, virus inactivation and pretreatment assays, no significant difference was observed between the treated and untreated groups.

### PRV gene expression was inhibited by Res

**Figure 4:**
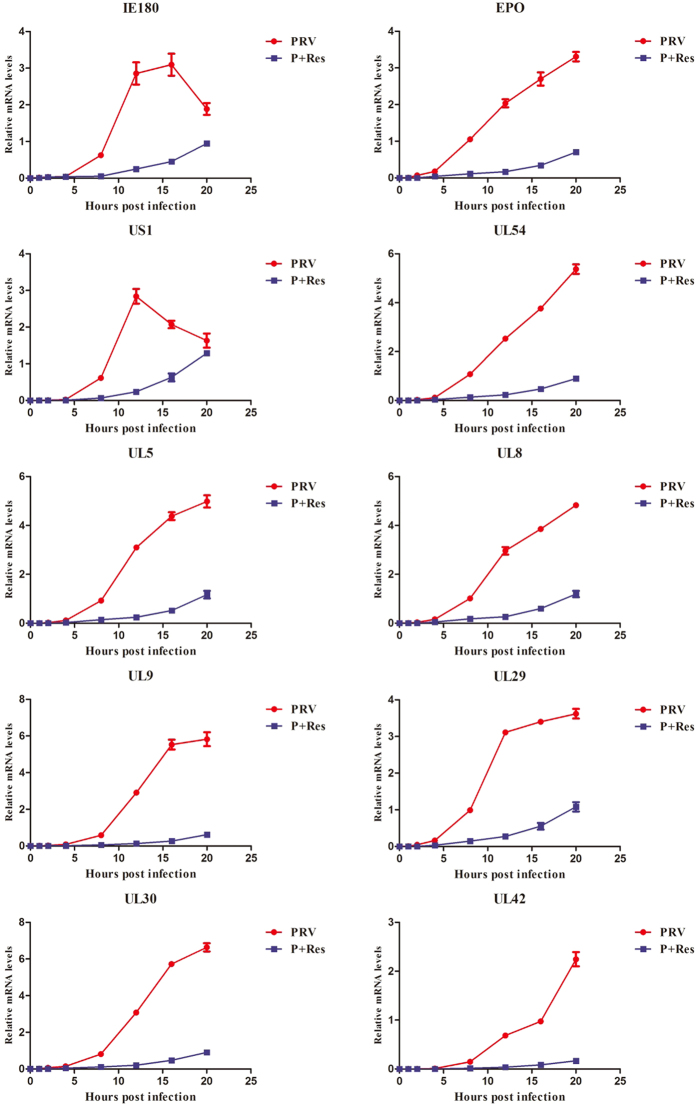
Res suppressed the expression of PRV genes. Gene expression levels of PRV in the presence or absence of Res (65.72 μM) were assayed at 0, 1, 2, 4, 8, 12, 16 and 20 hpi (n = 3, in each group).

The mode of action study indicated that the antiviral activity of Res contributed to blocking the stage of PRV replication. From the replication cycle of PRV, we know that PRV gene expression is the first step after the viral DNA passes into the cell nucleus^17^. Therefore, we detected the expressions of immediate-early gene, early genes and other genes essential for PRV replication. The expression levels of detected genes (IE180, EPO, US1, UL54, UL5, UL8, UL9, UL29, UL30 and UL42) are shown in Fig. 4. The expressions of all test genes in untreated cells began to significantly increase at 4 hpi and constantly grow until 16 hpi. However, Res obviously depressed this proliferation tendency. All gene expression was significantly suppressed within 20 hpi in the presence of Res (65.72 μM). These results indicated that the antiviral activity of Res was via inhibition of PRV gene expression.

### PRV-induced antiviral gene expression were inhibited by Res

**Figure 5:**
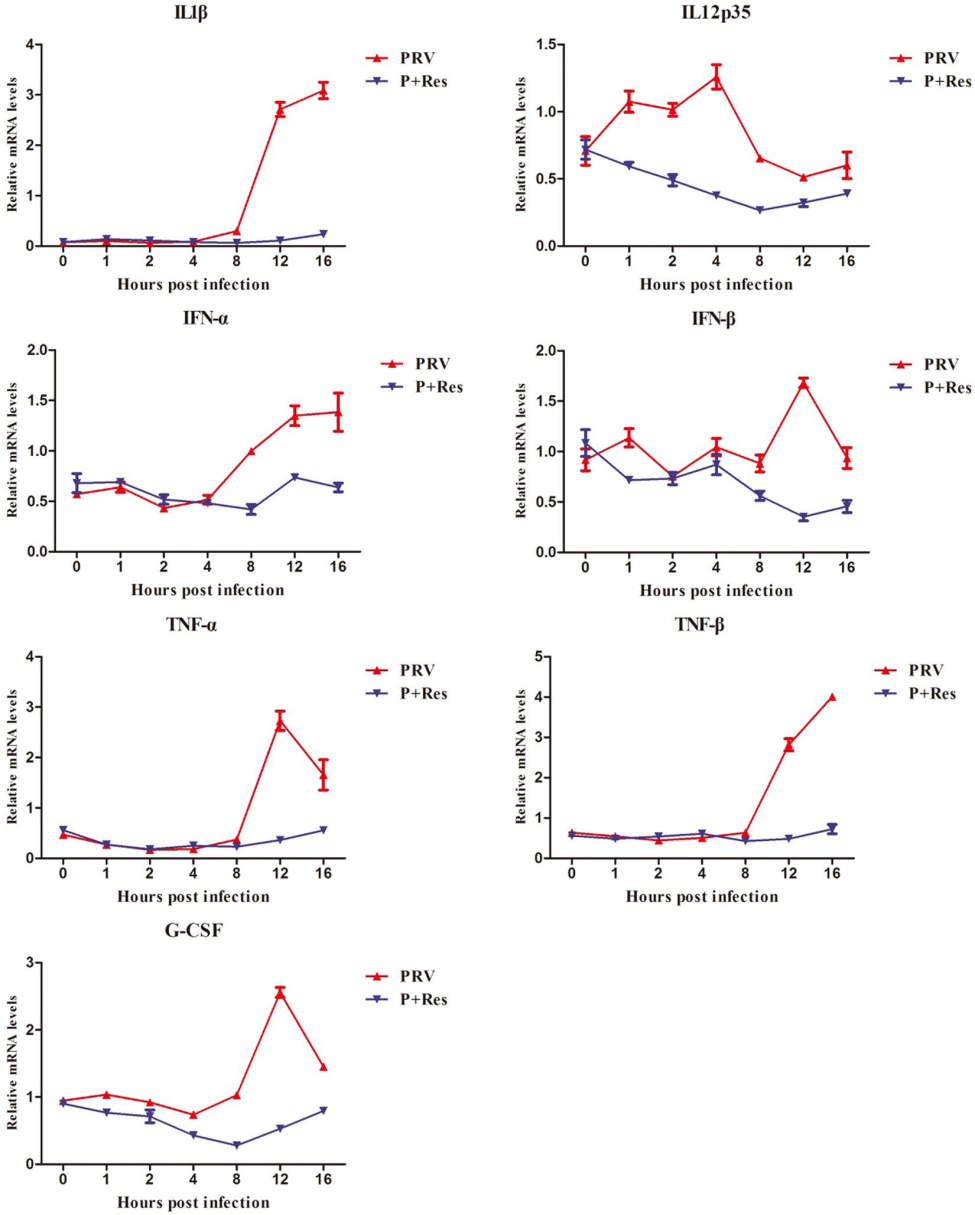
Res suppressed the expression of PRV-activated cytokine gene expression. Gene expression levels of activated cytokine genes in the presence or absence of Res (65.72 μM) were assayed at 0, 1, 2, 4, 8, 12 and 16 hpi (n = 3, in each group).

To determine whether the ability of Res to inhibit PRV gene expression also worked on PRV-induced gene expression in the host cell, we detected mRNA expression of antiviral cytokines in PK-15 cells. The results showed that there were seven antiviral genes that were activated by PRV, including IL-1β, IL-12p35, IFN-α, IFN-β, TNF-α, TNF-β and G-CSF (Table S3). After Res treatment, the seven genes were significantly inhibited, as shown in Fig. 5. These results indicated that the antiviral activity of Res was via inhibition of genes expression of both PRV and the host cells.

### Effect of Res on PRV activated cell signalling pathways

Cell signalling pathways are important for the expression of a wide variety of viral genes, and their activation is required for virus replication. In this study, we tested the activation of two typical cell signalling pathways (NF-κB and ERK1/2) for virus infection. The results showed that the NF-κB and ERK1/2 cell signalling pathways were activated by PRV infection in PK-15 cells, and Res could block the activation of NF-κB through inhibiting IκBα degradation and RelA nuclear translocation.

**Figure 6:**
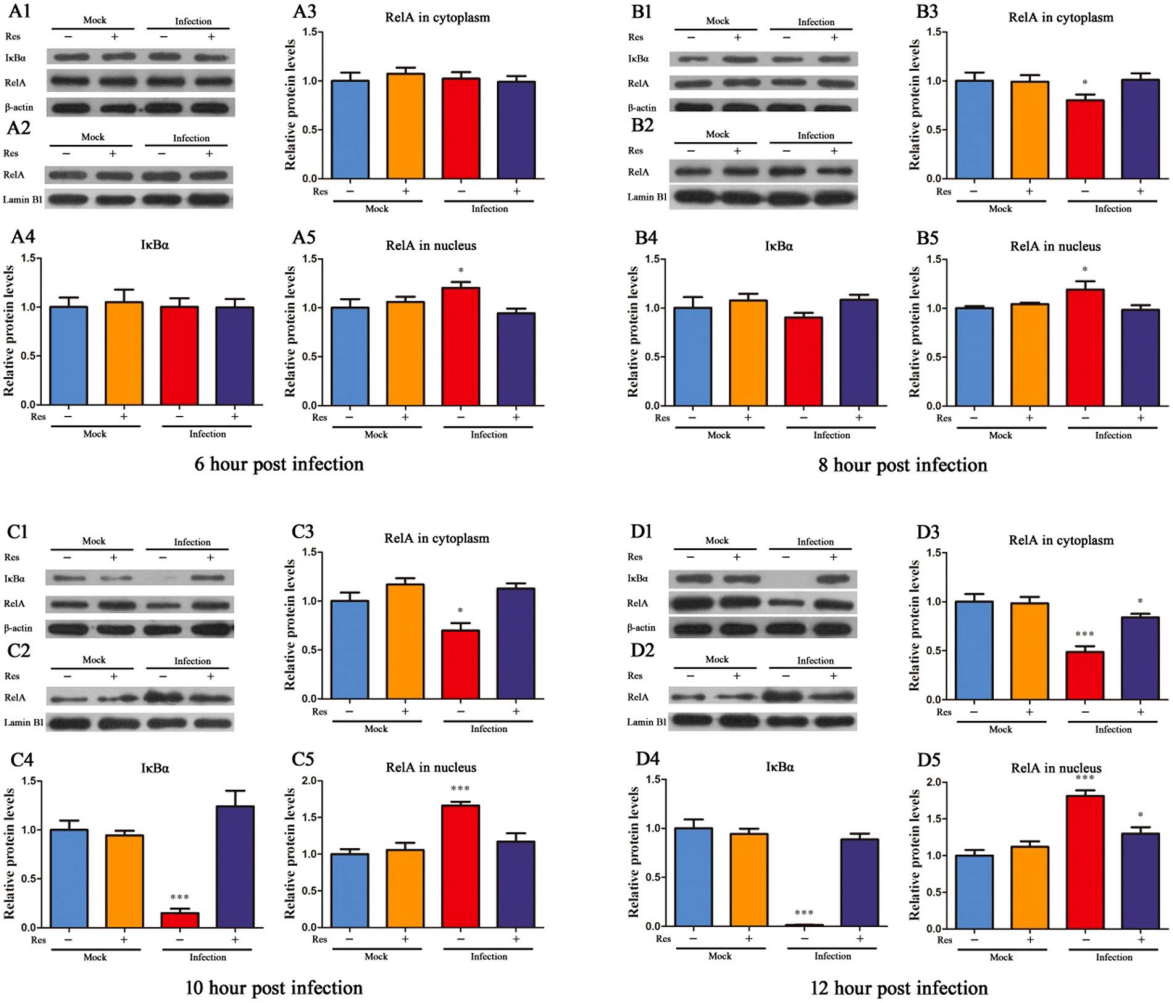
Res inhibited activation of NF-κB by PRV through inhibiting IκBα degradation and RelA nuclear translocation. PK-15 cells were infected with or without PRV at an MOI = 1 and with or without Res (65.72 μM). Expression of NF-κB signalling pathway proteins, IκBα, RelA and β-actin in cytoplasmic extracts and RelA and Lamin B1 in nuclear extracts were determined by Western blot at 0, 1, 2, 4, 6, 8, 10 and 12 hpi (there was no significant difference from 0 to 4 hpi in all groups, data not shown). Values are means ± SD (n = 3). Correlation analyses were evaluated by Pearson r2, ns: p > 0.05, *p < 0.05, **p < 0.01, and ***p < 0.001 vs. normal cells.

One of the key steps in activating the NF-κB signalling pathway is the degradation of the IκB (inhibitor of κB) kinases IκBα, IκBβ and IκBγ. Therefore, the relative levels of IκB kinases are widely used to determine whether the NF-κB signalling pathway is activated. In addition, the relative levels of RelA both in the cytoplasm and nucleus are also commonly used to evaluate the activation of the NF-κB signalling pathway. We detected the relative levels of IκBα in the PRV-infected PK-15 cells and the relative levels of RelA in both the cytoplasm and the nucleus. The results are shown in Fig. 6 (there were no significant differences from 0 to 4 hpi in all groups, data not shown). At 6 hpi, compared with normal cells, the relative levels of RelA in the nucleus of PRV-infected cells were significantly increased, while there were no significant differences between normal cells and PRV-infected cells with Res-treatment in the relative levels of RelA in the nucleus. At 8 hpi, compared with the normal cells, the relative levels of RelA in the cytoplasm of PRV-infected cells were significantly decreased, and the relative levels of RelA in the nucleus of PRV-infected cells were significantly increased, while there were no significant differences between normal cells and PRV-infected cells with Res-treatment regarding the relative levels of RelA in the nucleus and cytoplasm. At 10 hpi, compared with the normal cells, the relative levels of RelA and IκBα in the cytoplasm of PRV-infected cells were significantly decreased, and the relative levels of RelA in the nucleus of PRV-infected cells were significantly increased. There were no significant differences between normal cells and PRV-infected cells with Res-treatment regarding the relative levels of RelA in the nucleus and cytoplasm. At 12 hpi, compared to normal cells, the relative levels of RelA and IκBα in the cytoplasm of PRV-infected cells were significantly decreased, and the relative levels of RelA in the nucleus of PRV-infected cells were significantly increased. At the same time, the relative levels of RelA in the cytoplasm of PRV-infected cells with Res-treatment were significantly decreased, and the relative levels of RelA in the nucleus of PRV-infected cells with Res-treatment were significantly increased. There were no significant differences between normal cells and PRV-infected cells after Res-treatment regarding the relative levels of IκBα in the cytoplasm. These results indicated that PRV induced the degradation of IκBα and caused RelA translocation to the nucleus, where it binds to the promoter regions of NF-κB-responsive genes, resulting in increased gene expression. However, the degradation of IκBα and the translocation of RelA to the nucleus were significantly attenuated by Res.

Many transcription factors involved in a broad range of action have been shown to be phosphorylated and subsequently activated by ERK1/2, and the activation of ERK1/2 is achieved by autophosphorylation. Therefore, the activation of ERK1/2 reflects the activation of transcription factors, and we detected the relative levels of p-ERK1/2. The results are shown in Figure S3 (there was no significant difference from 0 to 4 hpi in all groups, data not shown). Compared to the mock without Res-treatment group, the relative levels of p-ERK1/2 were increased in the PRV-infected groups from 6 to 12 hpi. These results indicated that PRV could induce the phosphorylation of ERK1/2; nevertheless, Res had no significant influence on the PRV-induced phosphorylation of ERK1/2.

## Discussion

Despite that vaccines are wildly used in controlling PRV, outbreaks of PRV infection still occur^5^. Thus, effective control measures for PRV infection remain a major unmet challenge. Res, a member of the natural stilbene family, has been found to be effective on several viruses^1, 11, 18, 19^. In our previous study, Res showed potent antiviral activity against virulent duck enteritis virus both *in vitro* and *vivo* ^10, 16^. Although Res has been widely studied for its antiviral activities, research regarding its antiviral mechanisms remain lacking.

In this study, the cytotoxicity and antiviral effects of Res were confirmed by quantifying the cell viability with a CCK-8 kit, which has been widely used to determine the number of viable cells in recent research^20, 21^. Our results demonstrated that Res effectively inhibited the decreasing of cell viability induced by PRV infection in a dose-dependent manner. These results are similar to the research on antiviral activities of Res against African swine fever virus, Influenza A Virus, herpes simplex virus and human immunodeficiency virus-1^1, 11, 22, 23^. The antiviral activity of Res was also determined by detecting viral titres. PRV production in the presence of Res was significantly decreased in a dose-dependent manner. Vaccines are the only method of controlling PRV at present, but the recombination events resulting from different vaccine strains are important causes of morbidity and mortality^5, 6^. In addition, there is no drug available for treatment of PRV infection. Thus, Res is expected to be a new alternative control measure for PRV infection. This finding is consistent with the report that Res can attenuate the reproductive failure induced by PRV in mice^24^.

It has been reported that the antiviral activities of test samples were confirmed mainly based on their capability to reduce virus titre or inhibit CPE^16^. Real-time PCR based on TaqMan technology provides an accurate tool to quantify viral DNA. Our results confirmed that Res could reduce the production of PRV DNA. Next, we investigated the mode of action, and the results confirmed that the antiviral activity of Res was exerted through the inhibition of viral DNA replication, which is consistent with previous research^25^.

For the *alphaherpesvirinae* subfamily, in the early stage of infection, the viral genes are transcribed and some early viral proteins are expressed for further transcription and replication of the viral DNA^26^. The immediate-early gene, early gene and essential gene for replication of the viral DNA are essential for progeny virus production in PRV infection^17^. In this study, in order to determine the influence of Res on PRV gene expression, the PRV immediate-early gene (IE180), early gene (EPO, US1 and UL54) and seven essential genes for replication of the viral DNA (UL5, UL8, UL9, UL29, UL30, UL42 and UL52) were detected by FQ-PCR^17^. The results showed that the expression of detected genes were inhibited by Res, which confirmed the hypothesis that Res inhibits DNA replication by suppressing PRV gene expression. To determine whether Res inhibited antiviral gene expression activated by PRV infection, we detected the expression of seven activated genes by FQ-PCR. The results showed that the expression of activated genes were inhibited by Res. This finding is consistent with the report that Res could inhibit cyclic strain-induced endothelin-1 gene expression^27^. These results suggested that Res not only inhibited PRV gene expression, but it also inhibited the activated gene expression of host cells.

Gene expression requires activation of cell signalling pathways, and viruses can directly activate cell signalling pathways and utilize them in different ways^28^. Previous research found that HSV-1 and HSV-2 replication requires NF-κB and MAPK activation^29, 30^. To determine whether Res inhibited the activation of gene expression through influencing the activation of cell signalling pathways, Western blotting was used to detect the relative levels of proteins, which could reflect the activation of cell signalling pathways. The results showed that the ERK1/2 and NF-κB signalling pathways were activated by PRV infection in PK-15 cells. Res has been previously shown to inhibit ERK phosphorylation in smooth muscle cells^31^, but in our study Res did not significantly influence the activity of the ERK1/2 signalling pathway activated by PRV. Previous studies have shown that the chemopreventive properties of Res were associated with inhibition of activation of the IκB kinase^32^, and Res inhibited phorbol ester-induced expression of COX-2 and activation of NF-κB in mouse skin by blocking IκB kinase activity^33^. Similarly, our study showed that the activation of the NF-κB signalling pathway was significantly attenuated by Res through inhibiting the degradation of IκBα. A previous study showed that Res did not appear to directly block IKK activity^32^, so we did not detect the relative level of IKK. Our results indicated that Res was a potent inhibitor of IκBα degradation and RelA nuclear translocation. Thus, the inhibitory effect of Res on PRV-induced cell death and gene expression may be due to inhibition of the degradation of IκBα and attenuation of the activation of NF-κB nuclear translocation induced by PRV infection. This finding is consistent with the report that Res could inhibit the activation of the NF-κB cell signalling pathway during infection with Epstein Barr virus^8, 18^. These results indicate that the inhibitory effect of Res on herpesviruses may be due to the same molecular mechanism.

NF-κB is strongly associated with viral diseases^34–36^, and the activation of NF-κB is utilized by viruses to enhance their replication by increasing the efficiency of NF-κB-dependent viral gene transcription^37^. However, NF-κB activation has been shown to be blocked by antiviral compounds such as cyclopentenone prostanoids^37, 38^. Therefore, our results demonstrated that a potent antiviral compound, Res, also targets NF-κB activation to block both gene expression and the initiation of viral diseases.

**Figure 7:**
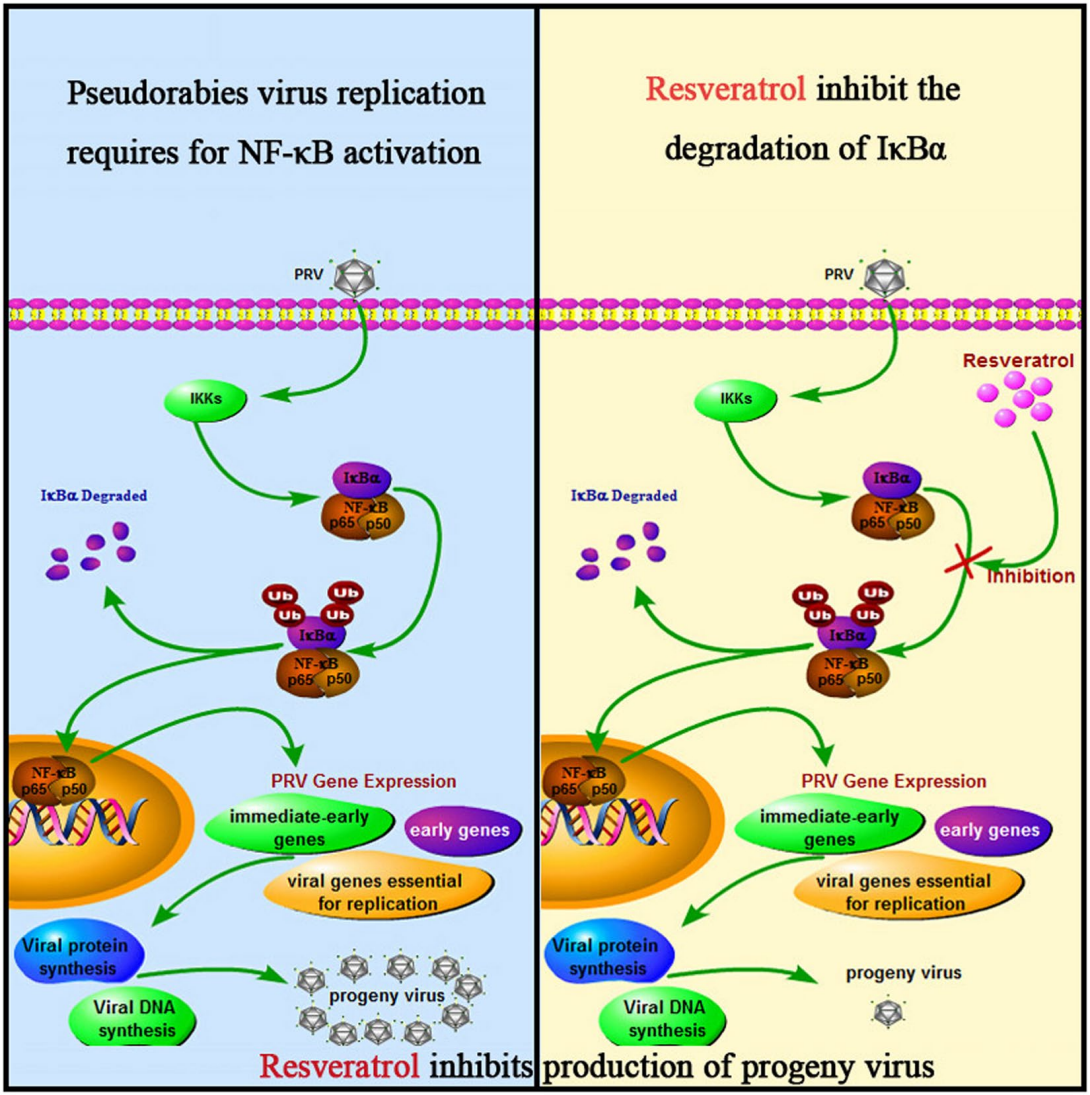
Schematic presentation of the Res anti-PRV molecular mechanism. The antiviral mechanism of Res against PRV attributes to inhibiting the degradation of IκBα kinase. Res could then block the expressions of NF-κB-dependent genes that would potentiate progeny virus production.

In summary, we report here the antiviral effect and molecular mechanism of Res against PRV in PK-15 cells. Our results demonstrated that Res could effectively inhibit PRV replication in cells. The antiviral mechanism of Res against PRV is summarized in Fig. 7, which was the inhibition of the degradation of IκBα kinase. Res blocked the expression of NF-κB-dependent genes that would potentiate progeny virus production. Thus, our results provide a molecular rationale to explain the broad antiviral properties of Res.

## Materials and Methods

### Compounds

Res (Figure S1), bought from Sigma with a purity of 98%, was prepared as a 45 mg/mL stock solution in ethanol and kept at −20 °C from light. For antiviral assays, Res was diluted to different concentrations in Dulbecco’s modified eagle medium (DMEM, HyClone).

### Cell Culture

The swine kidney cell line PK-15 (China Center for Type Culture Collection, Wuhan) was maintained in complete DMEM supplemented with 10% (v/v) foetal bovine serum (FBS, Gibco), 100 U/mL penicillin (HyClone), and 100 μg/mL streptomycin (HyClone). For the maintenance medium (MM), the serum concentration was reduced to 2%. Cells were incubated at 37 °C with 5% CO_2_.

### Virus Production and Infection

PRV (Rong A strain, China Veterinary Culture Collection Center) was propagated in PK-15 cells cultured in MM and then stored in our laboratory. PK-15 cells were grown to ∼90% confluence and infected with PRV at various MOIs. The mock-infected cells were treated with MM. After 1 h, the inoculum was removed by aspiration. Cells were then washed twice with PBS and incubated in MM at 37 °C for various times until harvesting.

### Cytotoxicity assay

The cytotoxic effect of Res on PK-15 cells was determined by quantifying cell viability using the cell counting kit-8 (CCK-8; Dojindo Molecular Technologies, Kumamoto, Japan). Briefly, cells in 96-well plates were exposed to different concentrations of Res in sextuplet. The test samples were suspended in MM (100 μL per well). After 48 h incubation, the CCK-8 reagent was added to the cultures according to the manufacturer’s instructions, and the plates were incubated for 1 h. Then, the absorbance values were measured using a microplate reader (Bio-Rad, USA) at 450 nm. The 50% cytotoxic concentration (CC_50_) was calculated as the compound concentration required for reducing cell viability by 50% according to the Reed-Muench method^39^.

### Effect of Res on cell viability in PRV-infected PK-15 cells

A 50 μL suspension containing 100 times 50% tissue culture infectious doses (TCID_50_) of virus and a serial two-fold dilution of Res solution were added to PK-15 monolayers in 96-well plates, and the plates were incubated at 37 °C for 1 h to allow virus attachment. Thereafter, the medium was aspirated to remove the unabsorbed virus. The cell monolayers were then washed with PBS, and MM containing serial two-fold dilutions of Res was added to the plates. The un-treated cells and un-infected cells were used as controls. The study was performed in sextuplet, and all plates were incubated at 37 °C in humidified 5% CO_2_. The virus-induced cytopathic effect (CPE) was observed daily by light microscopy in comparison with the controls. When the CPE of untreated cells was more than 75%, the CCK-8 kit described above was performed. The 50% effective concentration (EC_50_), which was estimated from the plots of the data, was the concentration that reduced 50% of cell death relative to that of the virus control.

### Effect of Res on virus titres

The PK-15 monolayers grown in 96-well plates were infected with PRV (MOI = 0.002) and then incubated in the presence or absence of Res according to the method described above. After incubation at 37 °C for 48 h, cells and media were harvested, and virus titres were determined by a TCID_50_ assay according to the Reed-Muench method^39^.

### Influence of Res on viral growth curve

Copies of viral genomes in the Res-treated cells were detected using the real-time quantitative PCR (FQ-PCR) method. Total DNA was extracted from PRV-infected cells using a Genomic DNA Extraction Kit (TaKaRa, Japan) and analysed using SsoAdvanced^TM^ Universal Probes Supermix (BIO-RAD, USA). The primers and probe were designed according to previous research^3^. The upstream and downstream primers were 5′-ACAAGTTCAAGGCCC ACATCTAC-3′ and 5′-GTCYGTGAAGCGGTTCGTG AT-3′, respectively, which were used to amplify a 95-bp fragment of the glycoprotein B gene of PRV (GenBank accession no. KJ526438). A 17-bp probe (5′-ACGTCATCGTCACGACC-3′) complementary to an internal region between two primers was selected and labelled with carboxyfluorescein at the 5′ end and with carboxytetramethylrhodamine at the -3′ end. The primers and probe were synthesized by the Beijing Genomics Institute (Beijing, China). The FQ-PCR was performed as one cycle of 120 s at 95 °C followed by 40 cycles of 5 s at 95 °C and 30 s at 56.5 °C. The number of gene copies in the reaction was deduced from the threshold cycle (CQ) values corresponding to the CQ values of the serial 10-fold dilutions of standard plasmids.

The PK-15 cells grown in 6-well plates were infected with PRV at an MOI = 0.01 in the presence or absence of Res for 1 h at 37 °C. MM with or without Res (65.72 μM) was added to the cultures after removal of the inoculums. The total DNA was extracted from PRV-infected cells at the indicated time points (3, 6, 9, 12, 15, 18, 21, 24, 27, 30, 33, 36, 39, 42, 45 and 48 h post infection [hpi]), and the copies of the PRV gene were detected by FQ-PCR assay.

### The mode of action assay

Virus inactivation assay: Equal volumes of Res solution and a concentrated virus suspension were placed in a tube for 1 h at 37 °C. The mixture without the test sample was used as a control. The infectious virus (MOI = 0.01) that remained in the drug was added to cell monolayers, and the cells were incubated for 1 h at 37 °C. MM was added after removal of the virus inoculums. After incubation for 42 h, the total DNA was extracted from PRV-infected cells, and copies of the PRV gene were detected by FQ-PCR assay.

Virus entry assay: The cell monolayers were infected with PRV at an MOI of 0.01 for 1 h at 4 °C. Viral inoculums were aspirated and washed twice to remove unabsorbed virus. Res was added to each well, and the plates were incubated at 37 °C for 1 h to allow entry. MM was added to the cultures after removal of viral inoculums. After incubation for 42 h, the total DNA was extracted from PRV-infected cells, and copies of the PRV gene were detected by FQ-PCR assay.

Pre-treatment assay: Res solutions were added to cell monolayers and incubated at 37 °C for 1 h. Then, Res was removed, and the monolayers were incubated with PRV at an MOI of 0.01 for 1 h at 37 °C. MM was added to the cultures after removal of the viral inoculums. After incubation for 42 h, the total DNA was extracted from PRV-infected cells and copies of PRV gene were detected by FQ-PCR assay.

Replication assay: The cell monolayers were infected with PRV at an MOI of 0.01 for 1 h at 37 °C. The cell monolayers were then washed with PBS and overlaid with Res solutions. The wells without Res were used as the control. After incubation for 42 h, the total DNA was extracted from PRV-infected cells, and copies of the PRV gene were detected by FQ-PCR assay.

### Gene expression analysis

A two-step FQ-PCR was used to test the inhibition effects of Res on the gene expression of PRV and antiviral cytokines in PK-15 cells. The cells grown in 6-well plates were infected with or without PRV at an MOI = 1 for 1 h at 37 °C. MM with or without Res (65.72 μM) was added to the cultures after removal of the inoculums. Cells were harvested for RNA isolation at 0, 1, 2, 4, 8, 12, 16 and 20 hpi. Total RNA was extracted from the cells using the RNAprep pure Cell Kit (Tiangen Biotech; Beijing, China) according to the manufacturer’s instructions. The RNA was immediately reverse transcribed into cDNA with the iSCRIPT cDNA SYNTHESIS Kit (Bio-Rad, USA) according to the manufacturer’s instructions.

FQ-PCR analysis was conducted in triplicate for each sample with a Bio-Rad CFX Connect Real-Time PCR System (Bio-Bad, USA) using the iQ SYBR Green Supermix kit (Bio-Bad, USA), and the primers are listed in the supplementary material (Table S2)^40, 41^. The PCR cycling was 3 min at 95 °C, followed by 40 cycles of 95 °C for 10 s (denaturation), 60 °C for 30 s (annealing), and 72 °C for 30 s (extension). A melting curve of the FQ-PCR products (55–95 °C) was also obtained to ensure the absence of artefacts. Expression of β-actin was used to normalize the differences in total cDNA levels in the samples. Data analysis was performed using Bio-Rad CFX Manager software.

### Western Blot assay

Proteins in the NF-κB and ERK1/2 signalling pathways, including IκBα, p-ERK1/2, and p65 (RelA), were examined by Western blot. The expression of β-actin and Lamin B1 were used to normalize the differences in total protein and nuclear protein, respectively. The PK-15 cells grown in a 25 cm^2^ flask were infected with or without PRV (MOI = 1) in the presence or absence of Res (65.72 μM) for 1 h at 37 °C. The cell monolayers were washed with PBS and MM with or without Res at a concentration of 65.72 μM. The total protein, cytoplasmic protein and nucleoprotein were extracted using a commercial kit (BOSTER, Wuhan, china) at 0, 1, 2, 4, 6, 8, 10 and 12 hpi, respectively. Lysates were mixed with Double Colour Protein Sample Loading Buffer (BOSTER, Wuhan, china) and heated at 95 °C for 5 min, followed by separation using SDS–PAGE under reducing conditions and blotting onto PVDF membranes (Bio-Rad). Proteins were stained using primary antibodies directed against IκBα (CST #4814), RelA (CST #6956), p-ERK1/2 (CST #4370), ERK1/2 (CST #4695), Lamin B1 (CST #13435), β-actin (CST #4970) and horseradish peroxidase-conjugated secondary antibody (CST #7074 or #7076). After the SuperSignal ECL reagent (Bio-Rad) was added, proteins were detected and band intensities were quantified using ImageJ.

### Statistical Analysis

All of the assays were performed in great than or equal to triplicate, and the mean values and standard deviation (SD) of the data were calculated. The statistical significance of the data was assessed using a two-tailed Student’s t test with GraphPad Prism software 5. Correlation analyses were evaluated by Pearson r^2^, ns: p > 0.05, *p < 0.05, **p < 0.01, and ***p < 0.001.

## Electronic supplementary material

Supplementary Information
