# Supplementary material for: Antiviral properties of resveratrol against pseudorabies virus are associated with the inhibition of IκB kinase activation
Source: Sci Rep. 2017 Aug 18;7:8782. doi: 10.1038/s41598-017-09365-0 (PMC5562710; doi:10.1038/s41598-017-09365-0)
Supplement: Supplementary file 1 — Supplementary Information [file 41598_2017_9365_MOESM1_ESM.doc]

**Antiviral properties of resveratrol against pseudorabies virus are associated with the inhibition of IκB kinase activation**

Xinghong Zhao ^a, 1^, Qiankun Cui ^a, 1^, Qiuting Fu ^a, 1^, Xu Song ^a, 1^, Renyong Jia ^b, *^, Yi Yang ^a^, Yuanfeng Zou ^a^, Lixia Li ^a^, Changliang He ^a^, Xiaoxia Liang ^a^, Lizi Yin ^a^, Juchun Lin ^a^, Gang Ye ^a^, Gang Shu ^a^, Ling Zhao ^a^, Fei Shi ^a^, Cheng Lv ^a^, Zhongqiong Yin ^1, *^

^a^ *Natural Medicine Research Center, College of Veterinary Medicine, Sichuan* *Agricultural University, Chengdu 611130, China;*

^b^ *Key laboratory of Animal Disease and Human Health of Sichuan Province, Sichuan Agricultural University, Chengdu 611130,China;*

^1^ *These authors contribute equally to this work.*

^*^ Corresponding author: Zhongqiong Yin

Postal address: Natural Medicine Research Center, College of Veterinary Medicine, Sichuan Agricultural University, Chengdu 611130, China

Tel.: +86 028 86290973; Fax: +86 028 86290973

E-mail address: yinzhongq@163.com (Z.Q. Yin)

Renyong Jia, E-mail address: cqrc_jry@163.com

**Supplementary Table S1. Effect of Res on viral life cycle**

| **Virus** | **Mode of action** | | **Reference** | **Virus** | **Mode of action** | **Reference** |
| --- | --- | --- | --- | --- | --- | --- |
| Adenovirus | Replication | 1 | | HIV-1 | Replication | 2 |
| DEV | Replication | 3 | | HIV-2 | Replication | 4 |
| EBV | Replication | 5 | | HTLV-1 | Transcription | 6 |
| HSV-1 | Replication/  entry | 7 | | ASFV | Replication | 8 |
| HSV-2 | Replication | 9 | | Influenza virus A | Replication | 10 |
| HCMV | Replication | 11 | | RSV | Replication | 12 |
| KSHV | Replication | 13 | | Human rhinovirus | Replication | 14 |
| VZV | Replication | 15 | | Vaccinia virus | Replication | 16 |
| Polyomavirus | Replication | 17 | | Calicivirus | Replication | 18 |

ASFV, African Swine Fever virus; DEV, Duck enteritis virus; EBV, Epstein–Barr virus; HCMV, human cytomegalovirus; HSV, herpes simplex virus; HTLV, human T-cell leukemia virus; KSHV, Kaposi’s sarcoma-associated herpesvirus; RSV, respiratory syncytial virus; VZV, varicella zoster virus.

**Supplementary Table S2.** Primer sequences used in this study ^a^

| **Name** | **Forward primer sequence (5'-3')** | **Reverse primer sequence (5'-3')** |
| --- | --- | --- |
| β-actin | GGACTTCGAGCAGGAGATGG | AGGAAGGAGGGCTGGAAGAG |
| ***PRV gene*** |  |  |
| IE180 | CATCGTGCTGGACACCATCGAG | ACGTAGACGTGGTAGTCCCCCA |
| EPO | GGGTGTGAACTATATCGACACGTC | TCAGAGTCAGAGTGTGCCTCG |
| US1 | AGCTCAACGAGCGCGACGTCTA | CGGAAGCTAAACTCGGACGCGA |
| UL54 | TGCAGCTACACCCTCGTCC | TCAAAACAGGTGGTTGCAGTAAA |
| UL5 | TGGACATGGCCACCTACGT | ACCGCGCGATGGTCAT |
| UL8 | CCGCTGATCCTGCCCTG | GAAGATGGGCTCCATGTGG |
| UL9 | CAAGTTCAAGCACCTGTTCGA | TGAGGCTGTCGTTGACGC |
| UL29 | CTGATCCTGCGCTACTGCG | ACTGCATCGTGATCCCCG |
| UL30 | TCATCACGAAGAAGAAGTACATCGG | CCTTCATGAGCATCTTGCCG |
| UL42 | GCTCCCCGAGCGTCG | CATGATGCAGTAGTCGTTGAACTC |
| UL52 | CGCGCAACTTTCACTTCCACGCA | TGCGCTCGAAGAAGCTCTCGTA |
| ***Cytokine*** |  |  |
| IL-1α | AGAATCTCAGAAACCCGACTGTTT | TTCAGCAACACGGGTTCGT |
| IL-1β | GCCCTGTACCCCAACTGGTA | CCAGGAAGACGGGCTTTTG |
| IL-2 | TGCAGCTCTTGTGTTGCATTG | CTTGAAGTAGGTGCACCGTTTG |
| IL-4 | CGTGACGGACGTCTTTGCT | CCCGGCAGAAGGTTTCCT |
| IL-5 | TGGTGGCAGAGACCTTGACA | CCATCGCCTATCAGCAGAGTT |
| IL-6 | TGGATAAGCTGCAGTCACAG | ATTATCCGAATGGCCCTCAG |
| IL-7 | CAGGGCACATTAACACTGTTCAA | CCAGGGAAGGTGGTTTTCTTC |
| IL-10 | CACAAGTCCGACTCAACGAA | GGGAAGTGGATGCAGCTGTT |
| IL-12p35 | AGTTCCAGGCCATGAATGCA | TGGCACAGTCTCACTGTTGA |
| IL-12p40 | ATGTCGTAGAGTTGGACTGG | ACTCTTTGACGTGGATGGTC |
| IL-18 | AGGGACATCAAGCCGTGTTT | CGGTCTGAGGTGCATTATCTGA |
| G-CSF | CGCCTGTAGCAGGGAGAAAA | GTAGGGGTTCACTCAGTGCC |
| IFN-α | CCCCTGTGCCTGGGAGAT | AGGTTTCTGGAGGAAGAGAAGGA |
| IFN-β | AGTTGCCTGGGACTCCTCAA | CCTCAGGGACCTCAAAGTTCAT |
| IFN-γ | ACTTATTTCTTAGCTTTTCAGCTTTGC | GGCGCCTGGCAGTAAGAG |
| TNF-α | CGACTCAGTGCCGAGATCAA | CCTGCCCAGATTCAGCAAAG |
| TNF-β | CACGGATCGTGCCTTCCT | GGACCAGCAGGGAGTTGTTG |

^a^ This table shows the list of primer pairs used for analysis of PRV and cytokine gene expression.

**Supplementary Table S3. Antiviral cytokine mRNA expressions in PRV infected PK-15 cells**

| **Cytokine** | **Result** | **Cytokine** | **Result** |
| --- | --- | --- | --- |
| ***Th-1 type*** |  | ***Chemokine*** |  |
| IL-1α | N.D. |  |
| IL-1β | Activation | G-CSF | Activation |
| IL-2 | N.D. |  |  |
| IL-6 | No influence | ***Th-2 type*** |  |
| IL-12p35 | Activation |  |
| IL-12p40 | N.D. | IL-4 | N.D. |
| IL-18 | No influence | IL-5 | No influence |
| IFN-α | Activation |  |  |
| IFN-β | Activation | ***Others*** |  |
| IFN-γ | N.D. |  |
| TNF-α | Activation | IL-7 | No influence |
| TNF-β | Activation | IL-10 | N.D. |

Analysis of cytokine expression in PK-15 cells infected with PRV by real-time quantitative PCR. N.D. means no mRNA detected.


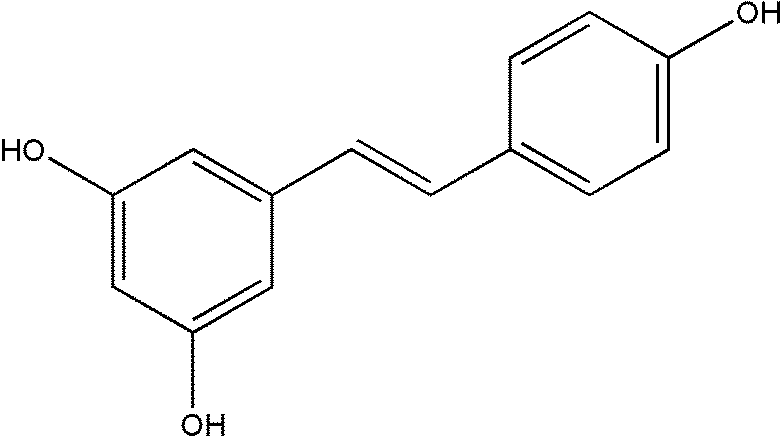


**Supplementary Figure S1. Chemical structure of Res.**


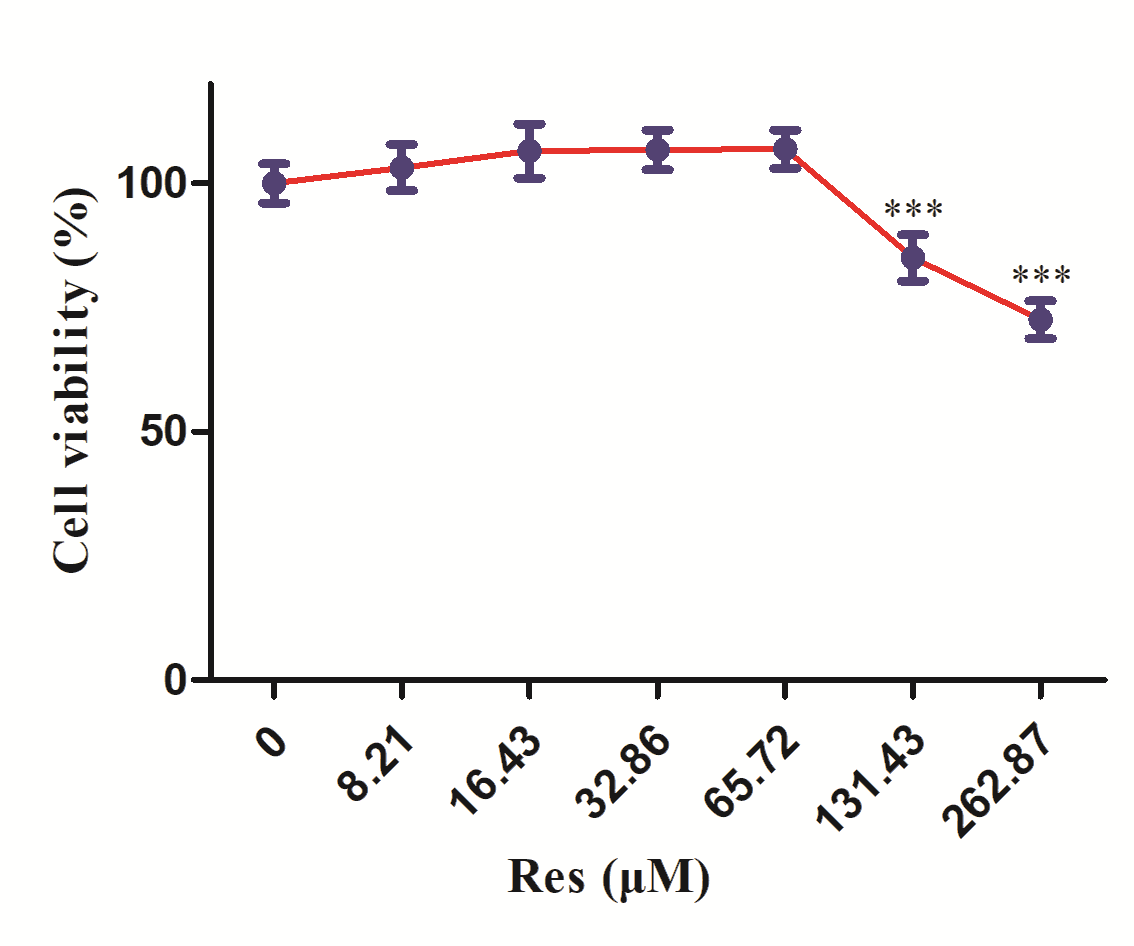


**Supplementary Figure S2. Cytotoxicity of Res.** The cell viability of Res in PK-15 cells, We tested a range of concentrations at which Res may exhibit potential cytotoxic activity on PK-15cells. After treatment for 48 h with Res, the number of viable PK-15 cells was determined using the CCK-8 kit. Values are means ± SD (n = 6). Correlation analyses were evaluated by Pearson r2, ns: p > 0.05, *p < 0.05, **p < 0.01, and ***p < 0.001 vs. normal cells.


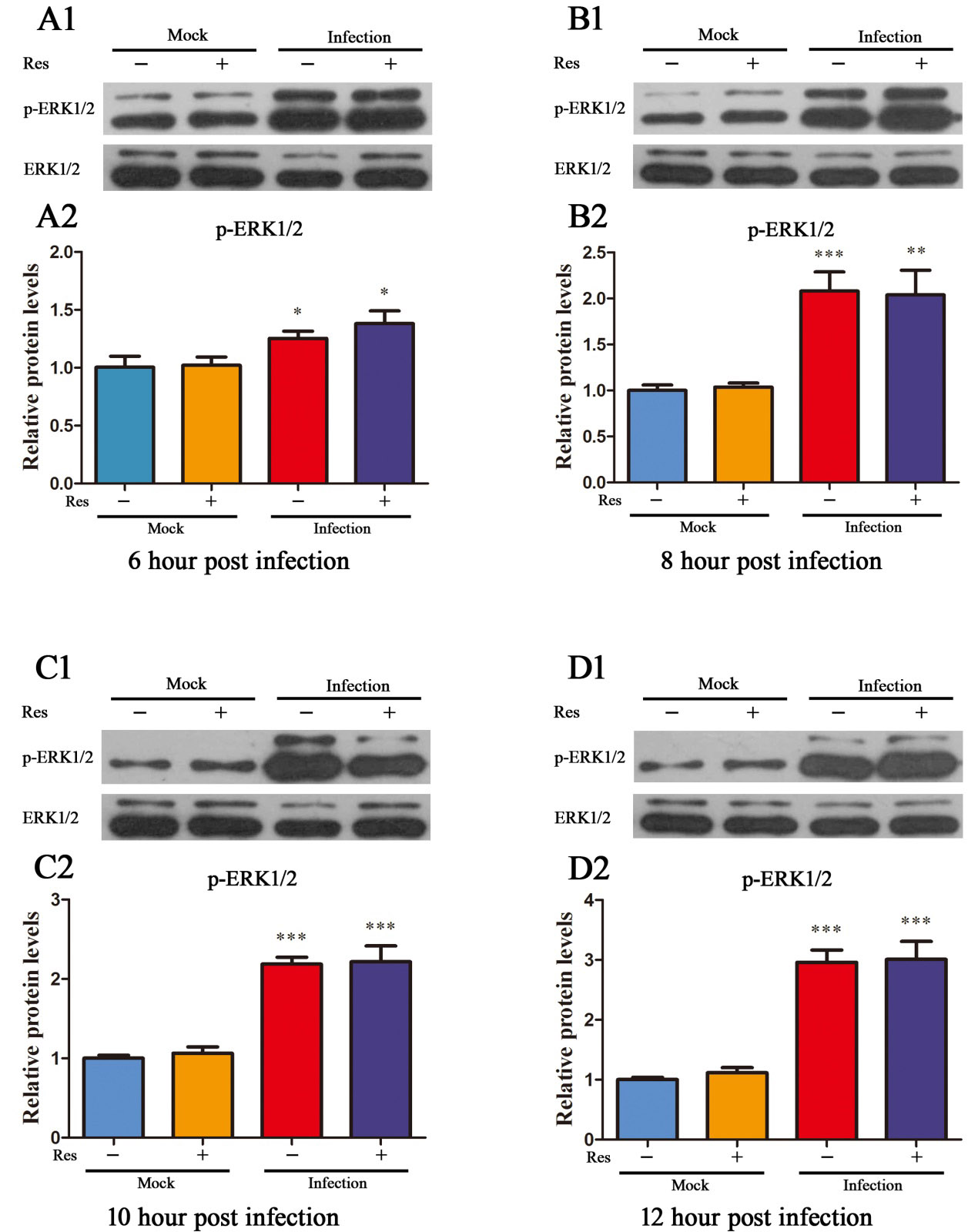


**Supplementary Figure S3. Res did not inhibit the phosphorylation of ERK1/2.** PK-15 cells were infected with or without PRV at an MOI=1 and with or without Res (65.72 μM). Expression of ERK1/2 signaling pathway proteins, ERK1/2 and p-ERK1/2 in total protein extracts were determined by western blot at 0, 1, 2, 4, 6, 8, 10 and 12 hpi (There was no significant difference from 0 to 4 hpi in all groups, data not shown). Values are means ± SD (n = 3). Correlation analyses were evaluated by Pearson r2, ns: p > 0.05, *p < 0.05, **p < 0.01, and ***p < 0.001 vs. normal cells.

**References**

1. Adam, V. et al. Synergistic and selective cancer cell killing mediated by the oncolytic adenoviral mutant AdDeltaDelta and dietary phytochemicals in prostate cancer models. Hum. Gene. Ther. **23(9)**, 1003-1015 (2012).

2. Heredia, A. et al. Targeting of the purine biosynthesis host cell pathway enhances the activity of tenofovir against sensitive and drug-resistant HIV-1. J. Infect. Dis. jit395 (2013).

3. Xu, J. et al. Inhibitory effect of resveratrol against duck enteritis virus in vitro. PloS One **8(6)**, e65213 (2013).

4. Beach, L. B., Rawson, J. M., Kim, B., Patterson, S. E., and Mansky, L. M. Novel inhibitors of human immunodeficiency virus type 2 infectivity. J. Gen. Virol. **95(12)**, 2778-2783 (2014).

5. Yiu, C. Y., Chen, S. Y., Chang, L. K., Chiu, Y. F., and Lin, T. P. Inhibitory effects of resveratrol on the Epstein-Barr virus lytic cycle. Molecules **15(10)**, 7115-7124 (2010).

6. Tang, H. M. V. et al. SIRT1 suppresses human T-cell leukemia virus type 1 transcription. J. Virol. **89(16)**, 8623-8631 (2015).

7. Leyton, L. et al. Nutraceutical activators of AMPK/Sirt1 axis inhibit viral production and protect neurons from neurodegenerative events triggered during HSV-1 infection. Virus Res. **205**, 63-72 (2015).

8. Galindo, I. et al. Comparative inhibitory activity of the stilbenes resveratrol and oxyresveratrol on African swine fever virus replication. Antivir. Res. **91(1)**, 57-63 (2011).

9. Docherty, J. J. et al. Resveratrol inhibition of herpes simplex virus replication. Antivir. Res. **43(3)**, 145-155 (1999).

10. Palamara, A. T. et al. Inhibition of influenza A virus replication by resveratrol. J. Infect. Dis. **191(10)**, 1719-1729 (2005).

11. Evers, D. L., Wang, X., Huong, S. M., Huang, D. Y., and Huang, E. S. 3, 4′, 5-Trihydroxy-trans-stilbene (resveratrol) inhibits human cytomegalovirus replication and virus-induced cellular signaling. Antivir. Res. **63(2)**, 85-95 (2004).

12. Xie, X. H. et al. Resveratrol Inhibits respiratory syncytial virus-induced IL-6 production, decreases viral replication, and downregulates TRIF expression in airway epithelial cells. Inflammation **35(4)**, 1392-1401 (2012).

13. Dyson, O. F., Walker, L. R., Whitehouse, A., Cook, P. P., and Akula, S. M. Resveratrol inhibits KSHV reactivation by lowering the levels of cellular EGR-1. PloS One **7(3)**, e33364 (2012).

14. Mastromarino, P. et al. Resveratrol inhibits rhinovirus replication and expression of inflammatory mediators in nasal epithelia. Antivir. Res. **123**, 15-21 (2015).

15. Docherty, J. J., Sweet, T. J., Bailey, E., Faith, S. A., and Booth, T. Resveratrol inhibition of varicella-zoster virus replication in vitro. Antivir. Res. **72(3)**, 171-177 (2006).

16. Cheltsov, A. V. et al. Vaccinia virus virulence factor N1L is a novel promising target for antiviral therapeutic intervention. J. Med. Chem. **53(10)**, 3899-3906 (2010).

17. Berardi, V., Ricci, F., Castelli, M., Galati, G., and Risuleo, G. Resveratrol exhibits a strong cytotoxic activity in cultured cells and has an antiviral action against polyomavirus: potential clinical use. J. Exp. Clin. Canc. Res. **28(1)**, 1 (2009).

18. Su, X., and D’Souza, D. H. Naturally occurring flavonoids against human norovirus surrogates. Food Environ. Virol. **5(2)**, 97-102 (2013).
